# Reliability and Validity of the MADM Scale in Measuring Maternal Autonomy During Hospital Births in Greece

**DOI:** 10.3390/healthcare14111587

**Published:** 2026-06-04

**Authors:** Eriketi Kokkosi, Antonis Galanos, Sofoklis Stavros, Efthalia Moustakli, Antigoni Sarantaki, Athina Diamanti, Kleanthi Gourounti, Maria Iliadou, Saraswathi Vedam, Angeliki Sarella

**Affiliations:** 1 Department of Midwifery, Faculty of Health and Caring Sciences, University of West Attica, 122 43 Athens, Greece; esarantaki@uniwa.gr (A.S.); adiamanti@uniwa.gr (A.D.); kgourounti@uniwa.gr (K.G.); miliad@uniwa.gr (M.I.); asare@uniwa.gr (A.S.); 2 Laboratory for Research of the Musculoskeletal System, School of Medicine, National and Kapodistrian University of Athens, 145 61 Athens, Greece; galanostat@yahoo.gr; 3 Third Department of Obstetrics and Gynecology, University General Hospital “ATTIKON”, Medical School, National and Kapodistrian University of Athens, 124 62 Athens, Greece; sfstavrou@med.uoa.gr; 4 Department of Nursing, School of Health Sciences, University of Ioannina, 4th Kilometer National Highway Str. Ioannina-Athens, 455 00 Ioannina, Greece; thaleia.moustakli@gmail.com; 5 Birth Place Lab, Division of Midwifery, Faculty of Medicine, University of British Columbia, Vancouver, BC V6T 1Z3, Canada; saraswathi.vedam@ubc.ca

**Keywords:** informed consent, midwifery-led care, obstetric care, perinatal period, patient autonomy, maternal rights, healthcare communication, autonomy, decision making, perinatal care

## Abstract

**Highlights:**

**What are the main findings?**

- The MADM scale is an effective instrument for assessing women’s autonomy in decision making within hospital childbirth settings.
- Autonomy is a key determinant of positive childbirth experiences.

**What is the implication of the main finding?**

- The application of the MADM scale may contribute to the development of quality indicators for respectful maternity care.
- The implementation of the MADM scale could support the implementation of patient-centered care models in different obstetric and midwifery settings.

**Abstract:**

**Background:** Autonomy in decision making is a critical component of respectful, person-centered maternity care. The Mother’s Autonomy in Decision Making (MADM) scale has been widely used internationally to evaluate women’s perceptions of autonomy during perinatal period. **Objectives**: To assess the reliability and validity of the Greek translation of the MADM questionnaire among postpartum women who have given birth in hospitals and clinics in Greece. **Methods**: A cross-sectional study was conducted with 450 postpartum women who gave birth in public and private maternity facilities in Greece. Participants completed the Greek MADM scale, and statistical analyses included exploratory and confirmatory factor analysis (EFA/CFA), internal consistency (Cronbach’s alpha), test–retest reliability (intraclass correlation coefficient, ICC), criterion validity (correlation with the Birth Satisfaction Scale (G-BSS), and Rasch analysis. **Results:** EFA revealed a one-factor structure explaining 77.6% of the variance (KMO = 0.913; Bartlett’s *p* < 0.001). CFA supported good model fit (CFI = 0.924, RMSEA = 0.085). The Greek MADM showed excellent internal consistency (Cronbach’s α = 0.951) and test–retest reliability (ICC = 0.995). Criterion validity was supported by moderate correlations with G-BSS subscales (r = 0.338–0.389, *p* < 0.005). Rasch analysis confirmed unidimensionality, high item reliability, and acceptable item fit. A cut-off score of 27.5 on the MADM scale was identified to distinguish between high and low–moderate birth satisfaction. **Conclusions**: The Greek version of the MADM scale demonstrates excellent psychometric properties and is a valid, reliable tool for assessing autonomy in maternity decision making. Its use can enhance quality assessment and promote respectful, autonomy-supportive perinatal care in Greek healthcare settings.

## 1. Introduction

In clinical practice, shared decision making (SDM) is now universally accepted as an intrinsic element of person-centered care and is linked to better patient outcomes even when the individual is not actively involved in the process [1]. During their engagement with health services, patients prefer to be active co-producers in decision making and intervention application [2]. They are the only constant across the continuum of care and can provide healthcare providers and systems with critical information to influence clinical decision making, drive system improvements, and support policy development. The routine inclusion of this rich perspective has the capacity to improve safety, responsiveness, and quality of healthcare services [3].

Within health psychology and person-centered care frameworks, autonomy is generally understood as an individual’s capacity and opportunity to make informed decisions regarding their healthcare based on personal values, preferences, and adequate information [4,5]. In maternity care, maternal autonomy extends beyond simple consent to include women’s perceived control over care processes, active participation in shared decision making, the ability to express preferences freely, and the experience of being respected and supported by healthcare professionals [6,7]. Thus, autonomy is conceptualized not only as decisional control but also as a relational and experiential construct shaped by the quality of communication, access to information, and provider–patient interactions [3]. The Mother’s Autonomy in Decision Making (MADM) scale operationalizes this construct by assessing women’s perceptions of inclusion, respect, understanding, and agency during maternity care decision-making processes [2].

In the context of maternity care, informed choice is an important aspect of respectful care. The National Institute for Health and Care Excellence (NICE) guideline “Intrapartum care for healthy women and babies” defines informed choice as the right of the woman to participate in decisions related to her care [4]. This right is part of larger patient rights structures, enshrined through national legislation and common law principles throughout Europe, and supplemented further through global human rights declarations (e.g., World Health Organization (WHO), 1994). Nevertheless, despite the robust framework of legislation, international research emphasizes continuing gaps between the establishment and practice of such rights, with most women reporting their autonomy being ignored when interacting with maternity care professionals [8].

According to research, the most prominent interconnected factors influencing women’s satisfaction with childbirth are their agency in decision making and the quality of their relationship with healthcare providers [9]. When women are empowered to engage actively in their care, this results in better outcomes, decreased healthcare expenses, and higher satisfaction rates for both women and care providers [7,10]. Still, too many women find themselves excluded from decision making, poorly advised on the options they have, and subjected to unwanted interventions against their personal values. These setbacks highlight the need to enhance communication between childbearing women and healthcare professionals [11,12].

In Greece, perinatal care is primarily delivered through public and private hospitals and clinics. This care model often involves women interacting with multiple healthcare professionals, particularly obstetricians and midwives, throughout pregnancy and childbirth. A smaller proportion of women with medically complex pregnancies may additionally receive care from fetal medicine specialists and other medical disciplines, including cardiologists, hematologists, and endocrinologists [13]. Only a small percentage of women in Greece choose home birth with independently practicing midwives, and these services are generally arranged privately outside the public healthcare system [14,15,16].

The organization of maternity services in Greece may also influence women’s experiences of autonomy and shared decision making. The involvement of multiple providers across different healthcare settings may contribute to fragmented continuity of care, inconsistent communication, and conflicting recommendations during pregnancy and childbirth [5,16]. Such fragmentation may create uncertainty or cognitive dissonance during the decision making process, potentially limiting women’s confidence, perceived control, and active participation in maternity care decisions. Despite the growing international emphasis on person-centered maternity care, there remains limited evidence regarding how women in Greece experience autonomy, informed consent, and involvement in decision making across hospital-based perinatal services [7,14].

Although previous Greek studies have explored childbirth satisfaction and women’s experiences of maternity care using instruments such as the Birth Satisfaction Scale (BSS), these measures do not specifically assess maternal autonomy as a distinct psychological and relational construct. General satisfaction scales and broader Patient-Reported Experience Measures (PREMs) primarily evaluate overall perceptions of care quality, emotional experience, or clinical environment, but may not adequately capture women’s perceived agency, decisional control, informed choice, or active participation in healthcare decision making [2,17]. Because maternal autonomy involves complex interpersonal and communication-related dimensions, its assessment requires dedicated and psychometrically validated instruments specifically designed to evaluate women’s experiences of inclusion, respect, understanding, and participation during maternity care interactions [18,19].

Existing evaluations of maternity care in Greece have predominantly focused on clinical indicators, such as cesarean section rates, intervention patterns, and perinatal outcomes, while comparatively less attention has been given to women’s reported experiences of communication, respect, informed choice, and shared decision making. PREMs are increasingly recognized as essential tools for assessing healthcare quality from the patient perspective. Because maternal autonomy is inherently relational and experiential, its assessment requires rigorously validated PREM instruments capable of generating reliable and comparable data across providers and care settings [18,20]. Within the Greek maternity care context, such measurement may support quality benchmarking, service evaluation, and policy initiatives aimed at strengthening respectful and autonomy-supportive maternity care. Therefore, the present study examined the psychometric properties of the Greek version of the MADM scale in a hospital-based maternity population [18,20].

Despite the increasing integration of informed consent principles into healthcare systems, the literature continues to identify important deficiencies in women’s experiences of autonomy and respectful maternity care. A systematic review conducted by Bohren et al. (2015) indicates that disrespect and abuse encountered during childbirth frequently include neglect of women’s autonomy [12]. The review identifies the need for appropriately valid and reliable instruments to measure mistreatment of women in childbirth. There has been incremental improvement (mainly in low-resource settings), yet only a small number of existing instruments have been designed collaboratively with service users and tested on large and diverse populations.

To explore women’s experiences of decision making in Greek maternity care, the present study validated and applied the Mother’s Autonomy in Decision Making (MADM) scale in a sample of Greek perinatal service users. The study was designed to evaluate the psychometric performance of the Greek MADM using complementary approaches derived from both Classical Test Theory (CTT) and Item Response Theory (IRT). Exploratory factor analysis (EFA) and confirmatory factor analysis (CFA) were conducted sequentially to examine and confirm the underlying dimensional structure of the instrument, while Rasch analysis was also applied to evaluate item functioning, unidimensionality, reliability, and measurement performance at the item and respondent levels. The combined use of CTT- and IRT-based methods provides a more comprehensive assessment of construct validity and measurement precision than either approach alone.

Based on previous international validations of the MADM scale, we hypothesized that the Greek version would demonstrate a unidimensional factor structure, high internal consistency, satisfactory test–retest reliability, and acceptable construct validity in relation to birth satisfaction measures. We further expected the scale to demonstrate satisfactory item fit and unidimensionality within the Rasch measurement framework. Through this validation process, the study aimed to establish the Greek MADM as a reliable and culturally appropriate PREM for assessing maternal autonomy and shared decision making within Greek maternity care settings.

## 2. Materials and Methods

We tested the psychometric properties of the Greek language version of the Mother’s Autonomy in Decision Making (MADM) scale, as relevant to Greek perinatal service users. The scale reflects the level to which midwives and obstetricians respect and empower women to take charge of their care decisions. Ethical approval was requested and obtained from the UNIWA (University of West Attica) Research Ethics Committee (approval number 57817/22-07-2024) prior to conducting the PhD study of the principal investigator (E.Κ.).

### 2.1. Study Measures

The MADM Scale was designed by Vedam et al. (2017) to evaluate women’s agency during the process of decision making in maternity care. The seven-item scale is a well-established and validated instrument in which women use a six-point Likert scale to reflect on their interactions during a decision-making process with maternity care providers (range of scores: 7–42) [21].

MADM showed evidence of construct validity and reliability, and the final Cronbach’s alpha for the subscales ranged from 0.90. Since its initial evaluation, MADM has been translated and used in numerous studies worldwide [21].

The Greek version of the Mother’s Autonomy in Decision Making (MADM) scale was embedded within a broader self-administered survey designed to capture sociodemographic information (age, marital status, education level, and employment status), obstetric history (parity, gestational age at birth), pregnancy risk status (self-reported low- or high-risk classification), and birth characteristics including mode of birth, onset of labour (spontaneous, induced, or scheduled caesarean), type of maternity facility (public or private), place of birth, and primary maternity care provider (obstetrician, midwife, or a shared model of care). The instrument also included the Greek version of the Birth Satisfaction Scale-Revised (G-BSS) to allow assessment of criterion validity. Prior to data collection, two stages of refinement were completed. Initially, the full survey was reviewed by a panel of senior midwifery academics and clinical experts to assess its face and content validity, wording clarity, and contextual appropriateness within the maternity services offered by Greece’s private and public hospital trusts. Minor linguistic refinements were made following this review. Thereafter, the survey was piloted with a group of postpartum women (*n* = 59) to evaluate comprehensibility and completion time, resulting in minor adjustments to item sequencing and wording. No substantive changes were made to the validated MADM items.

### 2.2. Participants and Eligibility Criteria

A convenience sample of 450 postpartum women was recruited between September 2024 and September 2025. Participants were women of reproductive age (18–49 years) who had given birth in public hospitals or private maternity clinics in Greece during the study period.

Eligible participants had experienced a low-risk pregnancy and birth, regardless of parity or mode of birth (vaginal birth or planned cesarean section), and were within the six-week postpartum period at the time of participation. For this initial psychometric validation study, the inclusion of women with self-reported low-risk pregnancies was intended to create a relatively homogeneous sample of postpartum women experiencing routine maternity care pathways. This approach was selected to minimize the potential influence of acute medical complexity, emergency obstetric interventions, or severe maternal distress on women’s responses during the validation phase of the instrument. Fluency in the Greek language was a prerequisite, as it was deemed necessary to obtain truly informed consent prior to completing the questionnaire.

Women were excluded from the analysis if they declined to provide informed consent, reported a high-risk pregnancy, had undergone an emergency obstetric delivery according to international clinical criteria, or reported childbirth experiences that occurred outside of Greece. Because pregnancy risk status was based on participant self-report, this classification may also have reflected women’s subjective perceptions of pregnancy complexity or vulnerability in addition to formal medical categorization.

Prior to statistical analysis, all questionnaires were screened for completeness. Because psychometric procedures such as factor analysis, reliability estimation, and Rasch modeling require complete item-level data for accurate parameter estimation and model stability [22,23], questionnaires with missing responses would have been excluded. However, all 450 questionnaires were completed and contained no missing item-level data. Consequently, no participants were excluded from the analyses, and no missing-data mechanism assessment was required.

According to the formula proposed by Viechtbauer et al., a minimum of 59 participants is required to detect potential issues with a 5% prevalence at a 95% confidence level [23]. The study’s achieved sample of 450 participants substantially exceeded this minimum requirement and was considered adequate for factor analysis, reliability testing, and validity assessment.

### 2.3. Study Procedures

Professor Vedam, the scale’s creator, granted permission to use the MADM scale via email and permitted the Greek translation by Serpetini (2024) for academic use. Approval was also given by Professor Hollins Martin via email for the use of the BSS-R scale translated into Greek by Vardavaki (2015) as the gold standard.

All respondents were informed about the voluntary and confidential nature of participation in the study. No identifiable data were collected, and participants were free to withdraw at any time without consequences. Approvals were obtained from the scientific councils of three maternity hospitals in the Attica region. To calculate test–retest reliability, 50 participants were asked to complete the questionnaire twice at 1-week intervals. According to the COSMIN guidelines, a sample size of at least 50 participants is considered methodologically adequate for evaluating stability and test–retest reliability [24,25].

### 2.4. Data Collection

The questionnaires were distributed in person and electronically following approval by the Research Ethics Committee of the University of West Attica (Ref. No. 57817/22-07-2024). The researcher (E.K.) approached and interviewed participants in person to complete the questionnaires at three public hospitals in the Attica region that approved the research process, respecting the privacy and sensitivity of the study and following the instructions and guidelines of the respective hospital committees. Women were approached at specific times and in designated departments, as indicated by hospital staff, in order to minimize discomfort or anxiety. Participation was completely anonymous, and no personal data capable of identifying participants were collected.

For the anonymous electronic questionnaires, the Microsoft Forms platform was used, as recommended by the University of West Attica (UNIWA) Research Ethics Committee, in accordance with General Data Protection Regulation (GDPR) compliance requirements. The sample of women was approached online via social media platforms (Facebook and Instagram). All data were stored on encrypted servers, and IP addresses or other identifying information were not collected.

The first page of the electronic questionnaire consisted of a separate participant information and informed consent page describing the study aims, the voluntary nature of participation, confidentiality procedures, and data protection measures. Participants were required to provide explicit electronic consent by selecting a checkbox indicating agreement to participate before accessing the questionnaire. If consent was not provided, the questionnaire did not proceed.

The anonymity of participants was fully guaranteed throughout the data collection, storage, and analysis processes, as all data were stored on encrypted servers, without the possibility of participant identification.

### 2.5. Statistical Analysis

The total sample was randomly divided into two independent subsamples. EFA using maximum likelihood extraction with Varimax rotation was conducted in the first subsample to examine the underlying factor structure of the seven MADM items. CFA was subsequently performed in the second independent subsample to cross-validate the factorial structure identified during the exploratory phase and to minimize potential overfitting bias. The selection of factors was based on the following criteria: (a) eigenvalues ≥ 1, (b) items with factor loadings > 0.40, and (c) items loaded only on one factor [26,27]. The number of factors to retain was also confirmed by using a Monte Carlo Principal Component Analysis (PCA) parallel analysis.

The CFA was carried out using the Analysis of Moment Structure (AMOS) Version 21 [28]. Given the seven-item structure of the MADM scale, the achieved subsample size exceeded commonly recommended participant-to-item ratios for confirmatory factor analysis [29].

Rejecting or accepting the model was based on several global fit indices: (a) chi-square-to-degrees of freedom (df) ratio; (b) the root mean square error of approximation (RMSEA); (c) the comparative fit index (CFI); (d) the normed fit index (NFI); (e) the goodness fit index (GFI); and (f) the adjusted GFI (AGFI). The chi-square-to-degrees of freedom (d. f.) ratio < 2.0 [29], RMSEA < 0.08 [30], CFI > 0.90 [30], GFI > 0.85 [31], AGFI > 0.80 [31], and NFI > 0.90 [32] indicate an acceptable fit.

Construct validity of the translated version of the MADM was assessed in two ways. The first, traditionally, was through convergent or criterion validity of the MADM questionnaire, determined by establishing its correlation with the Birth Satisfaction Scale (G-BSS) using Spearman’s correlation coefficient [33,34,35]. Moderate or high correlation between MADM scores and the established birth satisfaction measure were considered supportive of construct validity.

Secondly, construct validity was further examined using the Rasch measurement model, an Item Response Theory (IRT)-based approach. The combined use of Classical Test Theory and Rasch analysis was intended to provide a more comprehensive psychometric evaluation of the scale, including assessment of dimensionality, item functioning, measurement precision, and respondent reliability. The Rasch measurement model is one of the Item Response Theory models (IRT). IRT assumes that item characteristics are independent of respondents’ abilities and that respondents’ abilities are independent of the questionnaire items [35,36]. The validity of the questionnaire can be established from the perspectives of item and person reliability, item fit, and polarity using the Point Measure Correlation [37]. In addition, the Rasch Measurement Model provides empirical evidence of unidimensionality, which reflects the extent to which the instrument measures a single latent construct [36,38]. Rasch model analysis was carried out using the Winsteps version 3.68.0. In determining the validity of an instrument, item polarity, item fit, separation index, person reliability, and item reliability were evaluated [39].

The data were fitted to the Rasch Partial Credit Model (PCM). Although all questionnaire items used the same six-point Likert response format, they assessed conceptually different aspects of women’s decision-making experiences. Therefore, we did not assume that response category thresholds functioned identically across all items. The PCM was considered more appropriate than the Rating Scale Model (RSM) because it allows for item-specific threshold estimation and provides greater flexibility when category functioning varies between items.

For item polarity, the point measure correlation (PTMEA CORR) had to exceed 0, whereas for item fit, the infit and outfit Mean Square (MNSQ) values were required to fall within the range of 0.6 to 1.4 [35]. For separation values, all items were required to demonstrate values of ≥2.0 to indicate acceptable separation [35]. Lastly, both item reliability and person reliability values were required to exceed 0.8 [34].

Unidimensionality of a questionnaire indicates that the non-random variance in the data can be accounted for by a single underlying construct [37]. One method for assessing unidimensionality is principal component analysis (PCA) of residual scores, defined as the difference between the observed and expected scores [39]. The two PCA indicators used to assess unidimensionality were Raw Variance Explained by Measures (RVEM) and Unexplained Variance in the First Contrast (UVFC). RVEM values exceeding 40.0% were considered indicative of a strong principal measurement dimension [40,41], whereas UVFC values between 5 and 10% and an eigenvalue < 3.0 were considered supportive of unidimensionality [40,41].

The known-groups validity of the MADM questionnaire was examined according to the instrument’s ability to distinguish between subgroups of women based on the presence or absence of a birth plan. Birth plans are conceptually associated with greater engagement in shared decision making, communication with healthcare professionals, and expression of childbirth preferences, and were therefore expected to be associated with higher perceived autonomy during maternity care [4,42]. Independent samples *t*-tests were used for the statistical analysis.

Item analysis of the MADM questionnaire was performed by analyzing item discriminative power (corrected item-total correlation) and item decision-making indices (item mean divided by the total item score) using explanatory data analysis.

Interpretability refers to the degree to which qualitative meaning can be assigned to quantitative scores. It includes floor and ceiling effects as well as the minimal importance change (MIC). Floor or ceiling effects were considered present if more than 15% of respondents achieved the lowest or highest possible score, respectively [43]. The MIC was expressed as 0.5 × SD at baseline.

Measurement error refers to error in the score not attributed to the construct being measured and is expressed as the standard error of measurement (SEM), calculated using the formula: SEM = SD × √ (1 − ICC), where SD represents the standard deviation at baseline. The minimal detectable change (MDC) was calculated as SEM × 1.96 × √2 at the individual level.

Data distribution was evaluated. While the Kolmogorov–Smirnov test showed statistically significant deviations from normality (*p* < 0.05)—which is common in large samples—skewness and kurtosis indices fell within the acceptable ranges of −1 to +1. Consequently, the distributions were treated as approximately normal for subsequent analyses.

#### The Cut-Off Point of MADM Total Score

Receiver operating curve (ROC) analysis [44] was conducted to determine the cut-off level of the MADM total score for differentiating subgroups of women according to birth satisfaction, with standard errors and 95% confidence intervals (CI) estimated using the maximum likelihood estimation method. The optimal cut-off point was selected according to the maximization of sensitivity and specificity. The BSS score was used as the gold standard for birth satisfaction (BSS < 23 = low/moderate birth satisfaction vs. BSS ≥ 24 = high birth satisfaction). The cut-off value of 23 corresponded to 70% of the BSS score distribution [33,34].

Internal consistency reliability of the MADM was determined using Cronbach’s alpha coefficient [45]. A Cronbach’s α coefficient ≥ 0.7 was considered indicative of acceptable reliability for research purposes, whereas values > 0.8 were considered preferable for clinical applications [46].

Test–retest reliability (stability) was evaluated using the interclass correlation coefficient (ICC) [47] between the initial MADM assessment and reassessment after seven days. Because ICC does not account for systematic differences or agreement by chance, paired samples *t*-tests were additionally performed to assess systematic differences between the two assessments. Finally, the Bland–Altman plot [48,49] was used as a visual method for assessing agreement and stability.

The statistical analysis of the data was performed using SPSS version 21 (IBM Corporation, Somers, NY, USA), Winsteps Rasch Measurement Software version 3.81.0 (Winsteps, Beaverton, OR, USA), and the MedCalc^®^ Statistical Software version 20 (MedCalc Software Ltd., Ostend, Belgium). All tests were two-sided, and a *p*-value of <0.05 was considered statistically significant.

## 3. Results

### 3.1. Participants Characteristics

A total of 450 postpartum women were included in the study. For psychometric validation purposes, the total sample was randomly divided into two independent subsamples: 231 participants were allocated to exploratory factor analysis (EFA), whereas the remaining 219 participants were allocated to confirmatory factor analysis (CFA). Demographic and clinical characteristics of the EFA subsample are presented in Table 1.

### 3.2. EFA

EFA was conducted using maximum likelihood extraction with Varimax rotation. The Kaiser–Meyer–Olkin Measure of Sampling Adequacy was 0.913, indicating that the data were suitable for factor analysis. Bartlett’s test of sphericity demonstrated significant intercorrelations among items (χ^2^ = 1614.28, df = 21, *p* < 0.0001).

The seven MADM items loaded onto a single factor with an eigenvalue > 1, and all factor loadings exceeded the predefined threshold of 0.40. The eigenvalue for the first factor was 5.435, explaining 77.6% of the total variance. Factor loadings, representing the correlation coefficients between each item and the latent factor, ranged from 0.79 (item 1) to 0.93 (item 5) (Table 2).

**Figure 1:**
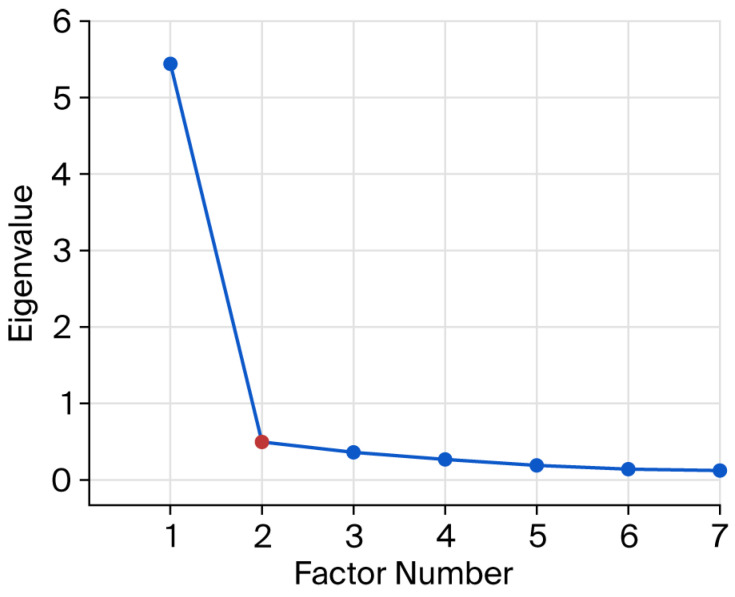
Scree plot of Exploratory Factor Analysis.

The scree plot (Figure 1) and Monte Carlo parallel analysis further supported a unidimensional structure. Specifically, the criterion value for the second eigenvalue obtained through parallel analysis (1.13) exceeded the observed eigenvalue of the second factor in the study data (0.49), supporting the retention of a single-factor solution.

### 3.3. CFA

**Figure 2:**
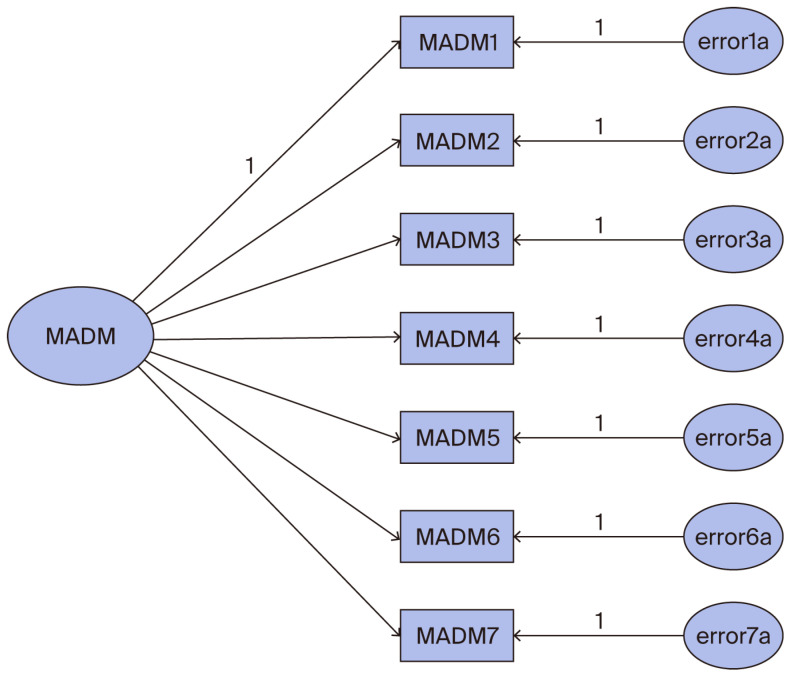
Confirmatory factor analysis of MADM structure.

The unidimensional model identified through EFA was subsequently examined using confirmatory factor analysis (CFA) in the second independent subsample (*n* = 219) (Figure 2). The CFA demonstrated acceptable model fit according to the predefined fit criteria. The resulting fit indices were: χ^2^ = 30.8, df = 14, χ^2^/df ratio = 2.2, RMSEA = 0.085, CFI = 0.924, NFI = 0.920, GFI = 0.860, AGFI = 0.780, and TLI = 0.892. Overall, these findings supported the adequacy of the single-factor structure identified during the exploratory phase and confirmed the unidimensional structure of the Greek MADM scale.

### 3.4. Convergent (Criterion) Validity

Convergent (criterion) validity was assessed by examining correlations between the MADM total score and the subscales of the Birth Satisfaction Scale (G-BSS). As presented in Table 3, moderate positive correlations were observed between the MADM total score and the G-BSS subscales of Stress Experienced (r = 0.389, *p* < 0.005), Quality of Care (r = 0.367, *p* < 0.005), and Women’s Attributes (r = 0.338, *p* < 0.005). These findings support the convergent validity of the Greek MADM scale, indicating that higher perceived autonomy in decision making was moderately associated with greater birth satisfaction.

### 3.5. Known-Groups Validity

The MADM total score successfully discriminated between subgroups of women based on the presence or absence of a birth plan. Women who reported having a birth plan demonstrated significantly higher MADM total scores compared with women who did not report having a birth plan (35.01 ± 8.95 vs. 27.19 ± 10.57, *p* < 0.005), supporting the known-groups validity of the instrument. These findings are consistent with the conceptual association among birth planning, active participation in maternity care, and greater perceived autonomy in decision making.

Although these findings support the construct validity of the MADM scale, the possibility that demographic or clinical differences between groups may have influenced the observed association cannot be fully excluded.

### 3.6. Item Analysis

Item analyses demonstrated autonomy decision-making indices (item mean divided by the total item score) ranging from 0.66 to 0.78 across the seven MADM items. The highest autonomy-related scores were observed for Items 2 and 5 (0.65), whereas the lowest score was observed for Item 7 (0.78).

**Figure 3:**
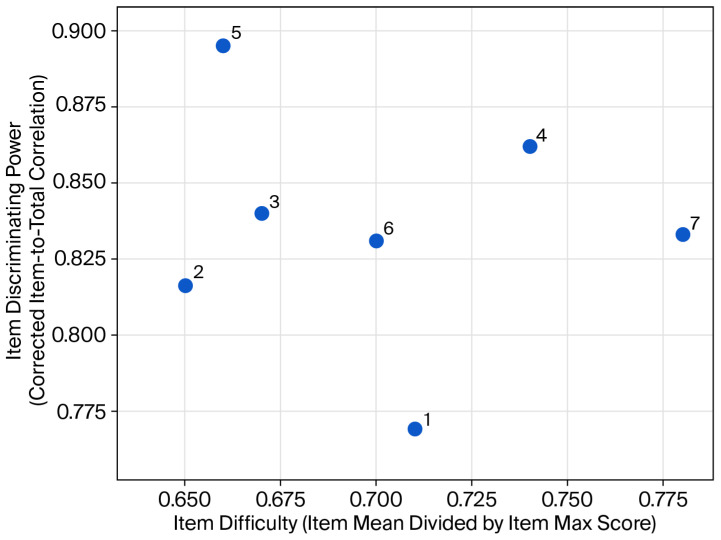
Item analysis of the MADM questionnaire.

Item discriminative power was evaluated using corrected item–total correlations based on Pearson’s product–moment correlation coefficients. Coefficients greater than 0.28 were considered indicative of satisfactory item discrimination. The discriminative indices of the MADM items ranged from 0.77 to 0.90, demonstrating strong discriminatory performance across all items. Item 5 demonstrated the highest discriminative capacity (r = 0.90), whereas Item 1 demonstrated the lowest, although still acceptable, discriminative value (r = 0.77) (Figure 3).

### 3.7. Interpretability (Floor or Ceiling Effects)

The proportion of participants scoring at the lowest possible level on the MADM total score was 3.5%, whereas 17% of participants achieved the highest possible score. Because the predefined threshold of 15% was marginally exceeded for the highest scores, a ceiling effect was considered present, whereas no floor effect was observed. The minimal important change (MIC) value was 5.35.

### 3.8. Measurement Error

The standard error of measurement (SEM) associated with the MADM total score was 1.02, indicating a low level of measurement error. The corresponding minimal detectable change (MDC) value was 2.83.

### 3.9. ROC Analysis and Cut-Off Determination

Receiver operating characteristic (ROC) analysis demonstrated that the *MADM total score* had acceptable discriminative ability in differentiating women by birth satisfaction levels. The area under the curve (AUC) was 0.729 (95% CI: 0.66–0.80, *p* < 0.005). The optimal cut-off point for the MADM total score was 27.5, corresponding to a sensitivity of 85% and a specificity of 55%.

**Figure 4:**
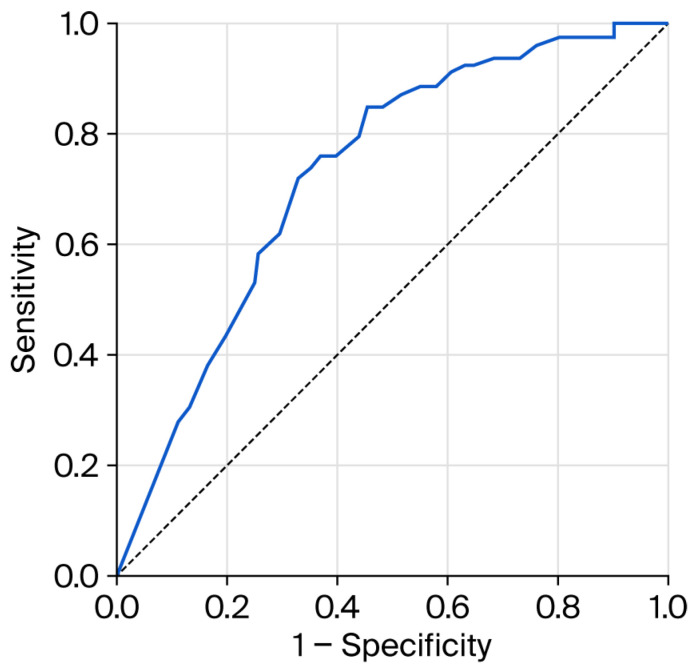
ROC analysis of the MADM questionnaire.

Women with MADM total scores above 27.5 were more likely to report high birth satisfaction, whereas women with scores below 27.5 were more likely to report low-to-moderate birth satisfaction (Figure 4).

### 3.10. Internal Consistency Reliability

The internal consistency of the MADM total score was assessed using Cronbach’s alpha coefficient and was estimated at 0.951, indicating excellent internal consistency reliability for the overall scale.

### 3.11. Test–Retest Reliability

The paired-samples *t*-test comparing the initial assessment and reassessment of the MADM total score demonstrated no statistically significant difference between the two measurements (73.75 ± 17.03 vs. 73.90 ± 16.56, *p* = 0.563). The intraclass correlation coefficient (ICC) between the initial assessment and reassessment was 0.995 (95% CI: 0.99–1.00, *p* < 0.005), indicating excellent test–retest reliability.

**Figure 5:**
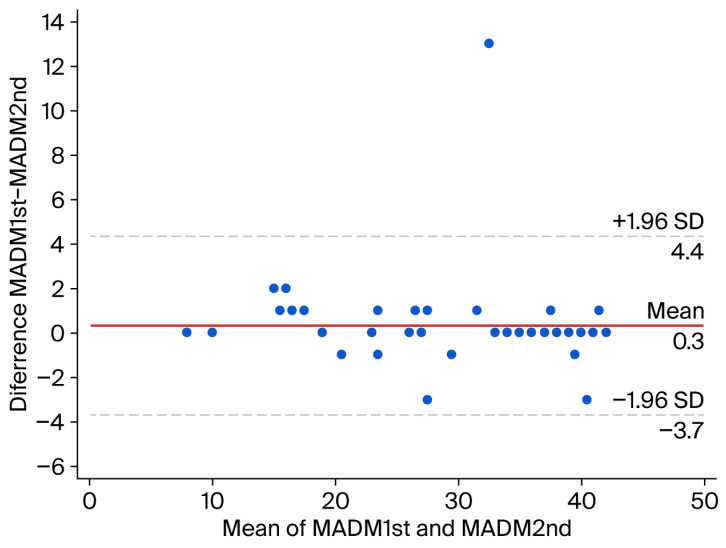
Bland–Altman plot of MADM total score mean difference: −0.2 (95% CI −3.3 to 3.0).

The Bland–Altman plot for the MADM total score is presented in Figure 5. Visual inspection of the scatterplot demonstrated that almost all differences were within the mean difference ± 2 standard deviations, further supporting agreement between the two assessments. Overall, these findings indicate excellent stability and temporal consistency of the MADM total score across the two assessment occasions.

### 3.12. Construct Validity of the MADM by the Rasch Model

The MADM questionnaire data were fitted to the Rasch partial credit model. Table 4 presents the item fit statistics and item difficulty hierarchy. The items were ordered according to their level of difficulty, indicating that, for this sample of participants, Item 2 represented the most difficult item, whereas Item 7 represented the easiest item to endorse.

The Greek MADM questionnaire demonstrated high reliability according to the Rasch model analysis, with satisfactory item and person reliability indices and acceptable separation indices.

The PTMEA CORR values, which assess item polarity, ranged from 0.80 to 0.90 across all seven items, indicating that all items were positively associated with the underlying construct and contributed appropriately to the measurement of maternal autonomy.

Only one item (Item 1) demonstrated infit (1.52) and outfit (1.50) mean square (MNSQ) values marginally exceeding the predefined acceptable range (0.6–1.4). However, because these deviations were minimal, the item was not considered to demonstrate substantial misfit.

The Greek MADM questionnaire also demonstrated good unidimensionality according to the Rasch analysis. Specifically, the Raw Variance Explained by Measures (RVEM) was 71.5%, exceeding the recommended threshold of 40%, whereas the Unexplained Variance in the First Contrast (UVFC) was 5.6% with an eigenvalue of 2.0, both within acceptable limits for unidimensional measurement. These findings indicate that the instrument measures a single underlying latent construct and that the individual items adequately fit the overall construct of maternal autonomy in decision making.

## 4. Discussion

This study evaluated the psychometric properties of the Greek version of the MADM scale in a sample of postpartum women who had given birth in Greek hospitals and maternity clinics. Overall, the findings provide strong evidence supporting the reliability, validity, and interpretability of the Greek MADM as a measure of women’s perceived autonomy in decision making during hospital-based maternity care.

The findings of both the exploratory and confirmatory factor analyses supported the unidimensional structure of the tool and were consistent with the original development study and previous international validation studies of the MADM scale [21]. Furthermore, the scale demonstrated excellent internal consistency (Cronbach’s α = 0.951) and excellent test–retest reliability (ICC = 0.998), supporting both the internal coherence and temporal stability of the instrument. Collectively, these findings indicate that the Greek MADM is a psychometrically robust instrument for assessing maternal autonomy within maternity care interactions.

Convergent validity was additionally supported through moderate positive correlations between the MADM total score and the dimensions of the Birth Satisfaction Scale (G-BSS), including stress experienced, quality of care, and women’s personal attributes. These findings suggest that greater perceived autonomy in maternity care is associated with more positive childbirth experiences and higher overall birth satisfaction. Similar associations between autonomy, respectful care, and childbirth satisfaction have also been reported in previous maternity care research and international MADM validation studies.

The Rasch analysis further supported the construct validity of the instrument by demonstrating acceptable item fit, satisfactory item polarity, strong reliability indices, and good unidimensionality. Together with the Classical Test Theory findings, these results suggest that the Greek MADM adequately captures a single underlying construct related to maternal autonomy in decision making.

The interpretability of the scale was also acceptable; however, a marginal ceiling effect (17%) was identified. This finding may indicate that a proportion of women in the study sample experienced genuinely high levels of perceived autonomy during maternity care interactions, particularly in supportive care environments. Alternatively, the ceiling effect may suggest that some MADM items are relatively easy to endorse, potentially limiting the sensitivity of the scale to discriminate between women reporting very high levels of autonomy. Although the ceiling effect only marginally exceeded the predefined threshold, future studies may further explore whether refinement or expansion of the scale could improve measurement precision at the higher end of the autonomy spectrum.

Known-groups validity was also confirmed through the scale’s ability to discriminate between women who reported having a birth plan and those who did not. Women with a birth plan demonstrated significantly higher MADM scores, supporting the sensitivity of the instrument to factors associated with greater engagement in shared decision making, antenatal planning, and communication with healthcare professionals.

A central principle of high-quality perinatal care is the preservation and promotion of women’s autonomy. Women who are adequately informed are better able to participate actively in decisions regarding their care and to select care options that align with their personal values and preferences [50]. In this context, maternal autonomy represents an important counterbalance to the dependency and passivity that may accompany highly medicalized models of childbirth care [51,52]. Supporting women’s autonomy, therefore, requires not only respectful interpersonal communication but also system-level investment in accessible, person-centered, and continuity-based maternity care services [53].

Equally important is women’s overall experience of care, including feeling emotionally respected, physically safe, adequately informed, and supported throughout pregnancy and childbirth. These dimensions are closely linked to the principles of informed consent and shared decision making, both of which are fundamental to ethical clinical practice and respectful maternity care. The contemporary emphasis on patient autonomy in healthcare emerged from broader civil rights, feminist, and social justice movements that challenged paternalistic models of authority and promoted individuals’ rights to participate actively in decisions affecting their lives and bodies [54].

Transparent communication regarding the risks and benefits of different maternity care options remains essential for authentic shared decision making and informed choice [7]. This is particularly relevant in contemporary maternity care settings, where increasing evidence continues to challenge historically dominant assumptions regarding the safety, appropriateness, and individualization of hospital-based childbirth practices [55].

### 4.1. Implications for Greek Maternity Care

The Greek maternity care system is characterized by a mixed public–private model of care provision and a relatively fragmented organization of maternity services. Women commonly receive care from multiple health professionals throughout pregnancy and childbirth, whereas continuity-of-care models led by midwives remain comparatively underutilized in routine clinical practice. Within this context, the findings of the present study suggest moderate-to-high levels of perceived maternal autonomy among participating women. More than half of the participants reported being asked about their preferred level of involvement in decision making, receiving adequate information regarding available care options, and being supported in understanding the advantages and disadvantages of different interventions or care pathways. Furthermore, a substantial proportion of women reported that their preferences were respected and that they played an active role in final decision-making processes.

Although these findings are encouraging, they also highlight the importance of systematically measuring maternal autonomy and women’s experiences of shared decision making within Greek maternity services. The Greek MADM scale offers healthcare providers, researchers, and policymakers a culturally and linguistically appropriate instrument for assessing women’s perceived involvement, respect, and autonomy during maternity care interactions. Its implementation within Greek maternity services may support the development of quality indicators for respectful maternity care and facilitate the identification of clinical settings or care processes in which women perceive insufficient participation in decision making.

Importantly, low MADM scores may help identify deficiencies in communication practices, informed consent procedures, support for women’s preferences, or opportunities for collaborative decision making during maternity care. Such findings could guide targeted quality-improvement initiatives, including training healthcare professionals in patient-centered communication, implementing structured informed decision-making protocols, expanding birth planning discussions, and developing continuity-of-care models that strengthen relational autonomy and patient engagement. Incorporating routine MADM assessment into routine maternity care audits and quality assurance frameworks may therefore promote more autonomy-supportive and respectful maternity care practices within Greek hospital settings.

Furthermore, the identified MADM cut-off score (27.5) may provide a preliminary practical reference point for evaluating interventions aimed at improving women’s childbirth experiences and perceived autonomy during maternity care.

### 4.2. Limitations

Several limitations of the present study should be acknowledged. First, the use of convenience sampling may limit the generalizability of the findings to the broader population of postpartum women in Greece. Although the sample included women from both public and private maternity settings and was considered adequate for psychometric validation purposes, future studies should aim to recruit more representative and geographically diverse populations, including women from rural regions and different socioeconomic and cultural backgrounds.

Second, the study population did not include women with high-risk pregnancies or emergency obstetric deliveries. Although this exclusion criterion was selected to create a relatively homogenous sample during the initial validation phase, it may limit the applicability of the findings to women experiencing medically complex pregnancies or urgent childbirth situations. Women with high-risk pregnancies may experience different levels of involvement in decision making and may face additional barriers to autonomy during maternity care. Further research is therefore needed to evaluate the validity and applicability of the MADM scale in high-risk and clinically diverse maternity populations.

Third, because pregnancy risk status was based on participant self-report, the classification of low- and high-risk pregnancies may have reflected both clinical characteristics and women’s subjective perceptions of vulnerability or pregnancy complexity. Consequently, some degree of misclassification cannot be excluded.

An additional limitation relates to the cross-sectional and immediate-postpartum nature of the assessment. The MADM questionnaire evaluated women’s perceived autonomy shortly after childbirth and therefore does not provide information regarding whether perceptions of autonomy change over time or whether they are associated with longer-term maternal outcomes, such as postpartum psychological well-being, maternal–infant bonding, or future healthcare engagement. Longitudinal studies are therefore warranted to investigate the temporal stability and predictive implications of maternal autonomy during maternity care.

Furthermore, the study did not include a formal assessment of social desirability bias. Participants may have overreported positive experiences of involvement in decision making or autonomy in order to align with perceived social expectations regarding maternity care experiences. This possibility may partially contribute to the observed ceiling effect identified in the present study. Future research should therefore consider incorporating measures of social desirability or response bias when evaluating maternal autonomy within maternity care settings.

Finally, although the MADM questionnaire captures women’s subjective experiences of autonomy and shared decision making, it does not directly assess actual clinical behaviors, provider communication practices, or the influence of institutional and organizational factors that may shape decision-making processes. Combining MADM findings with observational methods, provider-reported data, or qualitative approaches may provide a more comprehensive understanding of shared decision-making practices within Greek maternity care settings.

## 5. Conclusions

The Greek adaptation of the MADM scale demonstrated satisfactory psychometric properties, including strong reliability, validity, and unidimensionality, supporting its use as a measure of maternal autonomy in decision making among women giving birth in Greek maternity care settings. The availability of a culturally adapted Greek MADM scale may facilitate the evaluation of respectful, person-centered maternity care and support the development of interventions aimed at improving women’s involvement in decision making during pregnancy and childbirth.

In the context of ongoing discussions regarding maternity care reform in Greece, strengthening women’s autonomy, informed choice, and shared decision making should remain central to clinical practice, healthcare policy, and professional education.

## Figures and Tables

**Table 1:** Demographic and clinical characteristics of participants included in the exploratory factor analysis (EFA) subsample (*n* = 231). Abbreviation: SD, Standard Deviation.

Variable	Mean ± SD (Range)	Median (Range)	*n* (%)
Age (years)	33.40 ± 4.47 (23–48)		
Number of children		1.0 (1–5)	
Gestational week at birth	39.12 ± 1.18 (37–42)		
Family status; Married/Single			219 (94.8)/12 (5.2)
Education; High School/University/MSc–Ph.D.			35 (15.1)/88 (38.1)/108 (46.8)
Hospital; Public/Private			67 (29.0)/164 (71.0)
Mode of birth; Vaginal/Caesarean section			143 (62.0)/88 (38.0)
Onset of labour; Spontaneous/Induced/Scheduled caesarean section			94 (40.7)/99 (42.9)/38 (16.5)
MADM total	30.49 ± 10.71 (7–42)		

**Table 2:** Eigenvalues and explained variance of the MADM questionnaire.

Factors	Eigenvalues	% of Variance	Cumulative %
1	5.435	77.640	77.640
2	0.493	7.049	84.689
3	0.358	5.109	89.798
4	0.264	3.778	93.577
5	0.189	2.706	96.283
6	0.136	1.947	98.230
7	0.124	1.770	100.000
	**Items**	**Factor Loadings**	
	MADM5	0.926	
	MADM4	0.889	
	MADM3	0.869	
	MADM6	0.859	
	MADM7	0.852	
	MADM2	0.833	
	MADM1	0.785	

**Table 3:** Convergent (criterion) validity of the MADM questionnaire. Note: ** *p* < 0.005.

G-BSS Subscales	MADM Total Score
Stress Experienced	0.389 **
Quality of Care	0.367 **
Women Attributes	0.338 **

**Table 4:** Fit of admission MADM items to the Rasch model.

	Measure	SE	Infit MNSQ	Outfit MNSQ	PTMEA Corr
MADM2	54.36	1.42	1.26	1.22	0.85
MADM5	54.16	1.42	0.62	0.63	0.90
MADM3	53.36	1.41	1.07	1.10	0.86
MADM6	50.60	1.40	0.73	0.70	0.87
MADM1	48.07	1.40	1.52	1.50	0.80
MADM4	44.93	1.41	0.88	0.86	0.85
MADM7	44.53	1.41	0.84	0.93	0.85
**Criterion**		**Reliability**		**Separation**	
Person		0.91		3.12	
Item		0.87		2.59	
**Unidimensionality**					
Measures			Value		
Raw Variance Explained by Measure (RVEM)			71.5%		
Unexplained Variance in First Contrast (UVFC)			5.6%		
Eigenvalue of UVFC			2.0		

## Data Availability

The data supporting this research are available from the authors on reasonable request.

## Abbreviations

MADM	Mother’s Autonomy in Decision Making
BSS	Birth Satisfaction Scale
SDM	Shared Decision Making
EFA	Exploratory Factor Analysis
CFA	Confirmatory Factor Analysis
CTT	Classical Test Theory
IRT	Item Response Theory
ICC	Intraclass Correlation Coefficient
ROC	Receiver Operating Characteristic
PREM	Patient-Reported Experience Measure
